# Supplementary material for: Faecal microbiota transplantation for the treatment of diarrhoea induced by tyrosine-kinase inhibitors in patients with metastatic renal cell carcinoma
Source: Nat Commun. 2020 Aug 28;11:4333. doi: 10.1038/s41467-020-18127-y (PMC7455693; doi:10.1038/s41467-020-18127-y)
Supplement: Supplementary file 1 — Supplementary Information [file 41467_2020_18127_MOESM1_ESM.pdf]

Faecal microbiota transplantation for the treatment of diarrhoea induced by tyrosine-kinase inhibitors in patients with metastatic renal cell carcinoma

SUPPLEMENTARY INFORMATION

SUPPLEMENTARY FIGURES

Supplementary Figure 1. Effect sizes of comparisons between D-FMT patients and their respectives donors *vs* D-FMT patients and P-FMT patients. Effect sizes were calculated using Hedge's G statistic and vertical lines represent the 95% confidence interval of the statistic.

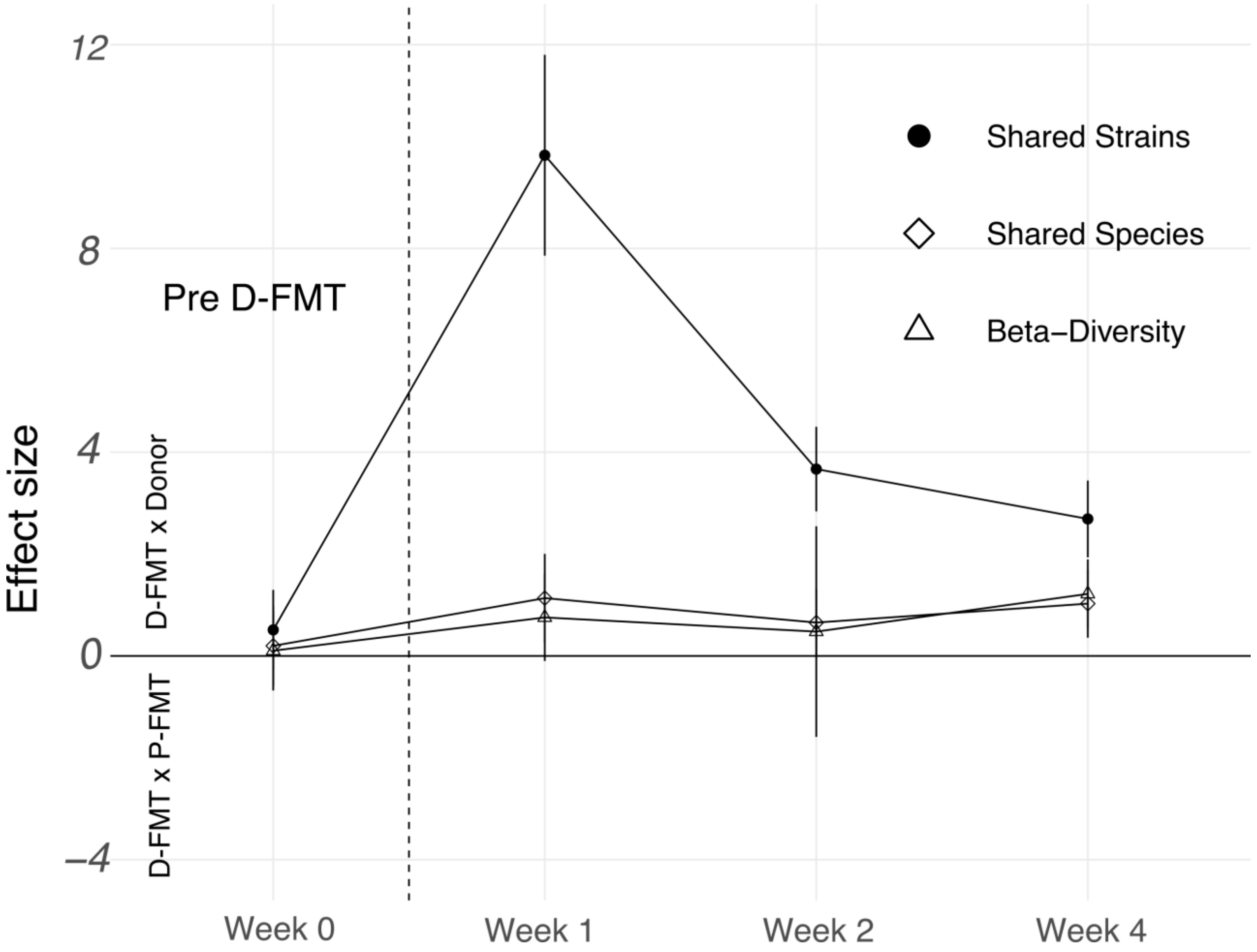

D-FMT= donor faecal microbiota transplantation; P-FMT= placebo faecal microbiota transplantation.

Supplementary Figure 2. Representative phylogenetic tree of *Akkermansia muciniphila* showing strain transmission for this species between D-FMT patients and their respective donors.

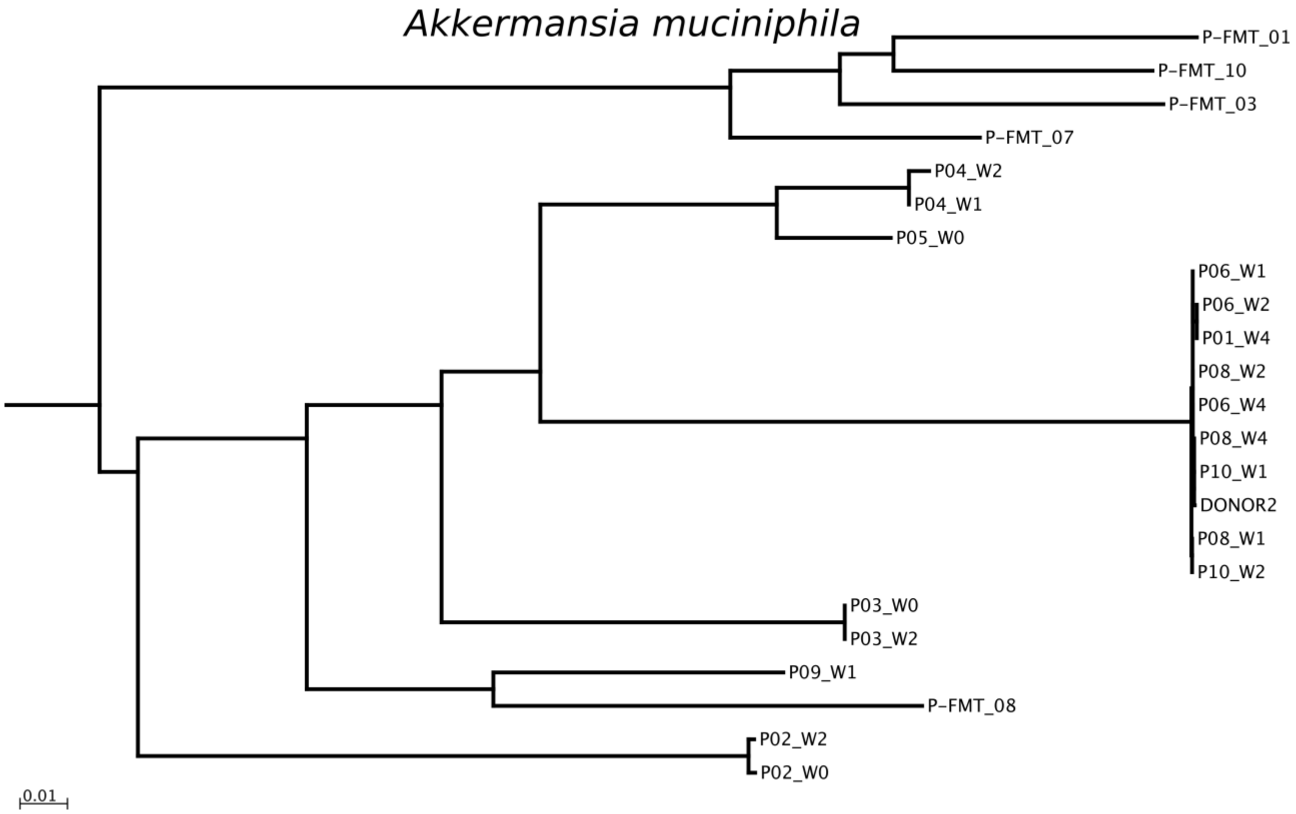

P= patient receiving donor faecal microbiota transplantation (D-FMT), W = Week. P-FMT= placebo faecal microbiota transplantation

Supplementary Figure 3. CONSORT Flow diagram of the subjects enrolled in the study.

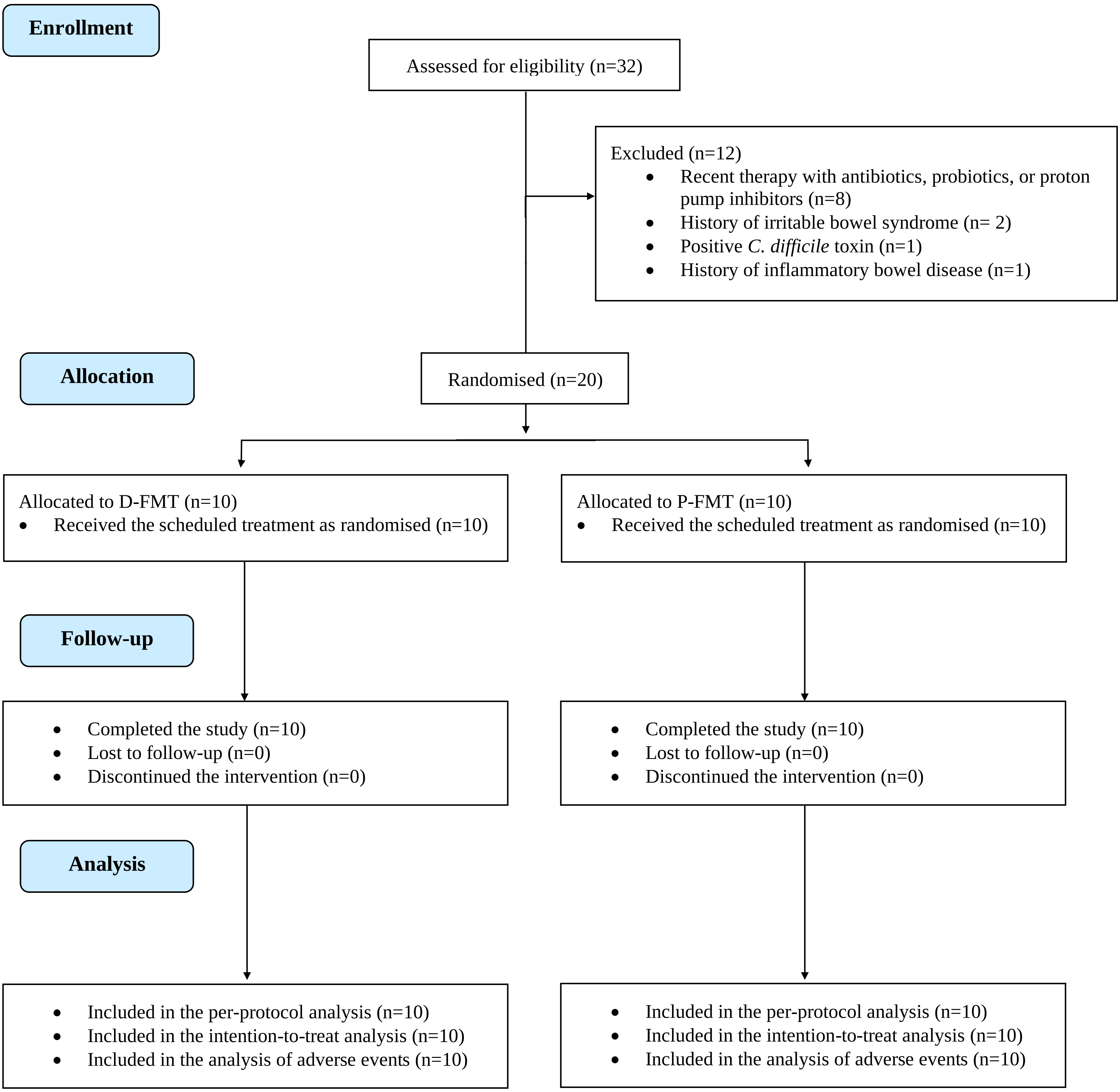

D-FMT= donor faecal microbiota transplantation; P-FMT= placebo faecal microbiota transplantation.

SUPPLEMENTARY TABLES.

Supplementary Table 1: Demographic and baseline clinical characteristics of enrolled patients

**Characteristic**	**D-FMT (N=10)**	**P-FMT (N=10)**	**P value***
Mean age in years (SD)	63.4 (9.38)	66.5 (12.74)	0.85
Gender (%)			1
Male	8 (80)	7 (70)	
Female	2 (20)	3 (30)	
BMI, kg/m2 (SD)	25 (3.74)	24.5 (3.47)	0.74
IMDC prognostic group (%)			1
Good	7 (70)	8 (80)	
Intermediate	3 (30)	2 (20)	
Poor	0	0	
Performance status**			1
0	8 (80)	7 (70)	
1	2 (20)	3 (30)	
Previous kidney surgery (%)			1
Yes	9 (90)	8 (80)	
No	1 (10)	2 (20)	
Site of metastases			
Lung	7 (70)	9 (90)	0.58
Liver	0 (0)	1 (10)	1
Pancreas	2 (20)	2 (20)	1
Lymph nodes	5 (50)	5 (50)	1
Bone	5 (50)	2 (20)	0.35
Cancer treatment (%)			0.6
Sunitinib	1 (10)	3 (30)	
50 mg, 4 weeks on/2 weeks off	1	2	
50 mg, 2 weeks on/1 week off		1	
Pazopanib	9 (90)	7 (70)	
800 mg daily	8	7	

600 mg daily	1		
Diarrhoea grade*** (%)			1
2	9 (90)	10 (100)	
3	1 (10)	0 (0)	

BMI= Body mass index; D-FMT= Donor faecal microbiota transplantation; IMDC: International metastatic renal cell carcinoma database consortium; P-FMT= placebo faecal microbiota transplantation; SD= standard deviation.

*Differences among groups were assessed with Wilcoxon-rank sum test for continuous data and with Fisher's exact probability test (using two-tailed P-values) for categorical data.
**According to the *Eastern Cooperative Oncology Group* (ECOG) scale
***According to the *Common Terminology Criteria (CTC) for Adverse Events* (AE) version 4.0. Only patients with grades 2 and 3 of diarrhoea were enrolled

Supplementary Table 2. CONSORT 2010 checklist of information to include when reporting a randomised trial

Section/Topic	Item No	Checklist item	Reported on page No
**Title and abstract**			
	1a	Identification as a randomised trial in the title	1
	1b	Structured summary of trial design, methods, results, and conclusions (for specific guidance see CONSORT for abstracts)	3
**Introduction**			
Background and objectives	2a	Scientific background and explanation of rationale	4
	2b	Specific objectives or hypotheses	4
**Methods**			
Trial design	3a	Description of trial design (such as parallel, factorial) including allocation ratio	10
	3b	Important changes to methods after trial commencement (such as eligibility criteria), with reasons	N.A.
Participants	4a	Eligibility criteria for participants	10-11
	4b	Settings and locations where the data were collected	10
Interventions	5	The interventions for each group with sufficient details to allow replication, including how and when they were actually administered	11-12
Outcomes	6a	Completely defined pre-specified primary and secondary outcome measures, including how and when they were assessed	14
	6b	Any changes to trial outcomes after the trial commenced, with reasons	N.A.
Sample size	7a	How sample size was determined	14-15
	7b	When applicable, explanation of any interim analyses and stopping guidelines	N.A.
Randomisation:			
Sequence generation	8a	Method used to generate the random allocation sequence	14
	8b	Type of randomisation; details of any restriction (such as blocking and block size)	14
Allocation concealment mechanism	9	Mechanism used to implement the random allocation sequence (such as sequentially numbered containers), describing any steps taken to conceal the sequence until interventions were assigned	14
Implementation	10	Who generated the random allocation sequence, who enrolled participants, and who assigned participants to interventions	14
Blinding	11a	If done, who was blinded after assignment to interventions (for example, participants, care providers, those assessing outcomes) and how	14
	11b	If relevant, description of the similarity of interventions	14
Statistical methods	12a	Statistical methods used to compare groups for primary and secondary outcomes	14-15

	12b	Methods for additional analyses, such as subgroup analyses and adjusted analyses	15
**Results**			
Participant flow (a diagram is strongly recommended)	13a	For each group, the numbers of participants who were randomly assigned, received intended treatment, and were analysed for the primary outcome	Supplementary material, page 4
	13b	For each group, losses and exclusions after randomisation, together with reasons	N.A.
Recruitment	14a	Dates defining the periods of recruitment and follow-up	4
	14b	Why the trial ended or was stopped	N.A.
Baseline data	15	A table showing baseline demographic and clinical characteristics for each group	Supplementary material, page 5
Numbers analysed	16	For each group, number of participants (denominator) included in each analysis and whether the analysis was by original assigned groups	Supplementary material, page 4
Outcomes and estimation	17a	For each primary and secondary outcome, results for each group, and the estimated effect size and its precision (such as 95% confidence interval)	6
	17b	For binary outcomes, presentation of both absolute and relative effect sizes is recommended	N.A.
Ancillary analyses	18	Results of any other analyses performed, including subgroup analyses and adjusted analyses, distinguishing pre-specified from exploratory	6-8
Harms	19	All important harms or unintended effects in each group (for specific guidance see CONSORT for harms)	N.A.
**Discussion**			
Limitations	20	Trial limitations, addressing sources of potential bias, imprecision, and, if relevant, multiplicity of analyses	9
Generalisability	21	Generalisability (external validity, applicability) of the trial findings	9
Interpretation	22	Interpretation consistent with results, balancing benefits and harms, and considering other relevant evidence	9
**Other information**			
Registration	23	Registration number and name of trial registry	10
Protocol	24	Where the full trial protocol can be accessed, if available	10
Funding	25	Sources of funding and other support (such as supply of drugs), role of funders	N.A.

SUPPLEMENTARY NOTE 1

STUDY PROTOCOL

FAECAL MICROBIOTA TRANSPLANTATION FOR THE TREATMENT OF DIARRHOEA
INDUCED BY TYROSINE-KINASE INHIBITORS IN PATIENTS
WITH METASTATIC RENAL CELL CARCINOMA

Table of Contents

1. Introduction and Study Rationale 3
2. Objectives 5
3. Methods 6
3.1 Study Design 9
3.2. Study Population 14
3.2.1 Inclusion criteria 14
3.2.2 Exclusion criteria 14
3.3. Baseline assessments 14
3.4. Treatments 10
3.4.1 Selection of stool donors 14
3.4.2 Manufacturing of faecal infusates 14
3.4.3 FMT procedures 14
3.5. Follow-up 10
3.6. Study Outcomes 13
3.6.1. Primary outcome 13
3.6.2. Secondary outcome 13
3.7. Randomization and blinding 20
3.8 Gut microbiota analysis 16
3.9 Sample size 16
3.10 Statistical Analysis 16
4. Safety Reporting 22
5. Ethics 23
6. References 23

1. INTRODUCTION AND STUDY RATIONALE

Despite the improvement in diagnosis and management, renal cell carcinoma (RCC) remains one of the most burdensome urological cancers, being the sixth most common malignancy in men and the 10th in women, accounting, respectively, for 5% and 3% of all cancers (1). Moreover, the incidence of RCC is increasing, especially in Western countries (2), accounting for nearly 60000 new cases per year in the United States (3). A considerable proportion of patients present with metastatic disease at diagnosis (4,5), and there are more than 140000 RCC-dependent deaths per year worldwide according to the World Health Organization (6).

Sunitinib and pazopanib are oral multi-targeted receptor tyrosine kinase inhibitors (TKIs) that have dramatically improved the survival of patients with metastatic RCC (7), and are commonly used as first-line option for this condition (8).

However, long-term use of these drugs is prevented by the development of toxicity. Diarrhoea is one of the most common side effects of TKIs, occurring in nearly 50% of patients (9-11). It decreases the quality of life of these patients, and often requires dose reduction and drug discontinuation (12), potentially decreasing the efficacy of TKIs.

To date there are no standardised strategies for TKIs-related diarrhoea, and current recommendations are supported by few evidence or real-life experience. Recommended treatment options include anti-motility agents, which are not targeted to act on the pathogenic pathways of diarrhoea (13).

Increasing evidence suggests that gut microbiota could influence the development of TKIs-induced diarrhoea. Overall, chemotherapy is known to drive, through the development of mucositis, deep compositional and functional alterations of gut microbiota (14). Mucositis occurs commonly after treatment with TKIs (15), and a specific dysbiotic profile has been found in patients with TKIs-induced diarrhoea (16).

In theory, the therapeutic modulation of gut microbiota could be an approach to alleviate TKI-induced diarrhoea. Although probiotics have been suggested as a possible treatment option for this condition, few evidence supports this indication (17,18).

Faecal microbiota transplantation (FMT) is the infusion of faecal microbiota from a healthy donor in the gut of a recipient with the aim of curing a specific disease. It has been increasingly recognised as a highly effective treatment against recurrent *C. difficile* infection (19,20).

FMT has been also examined as a potential approach for other disorders associated with a disruption of gut microbiota, including ulcerative colitis (21) or metabolic syndrome (22).

To date, the effects of FMT on chemotherapy-related diarrhoea are unknown. The aim of our study is to investigate the efficacy of faecal microbiota transplantation (FMT), compared with placebo FMT, in treating TKI-induced diarrhoea in patients with metastatic RCC.

2. OBJECTIVES

- To assess the efficacy of donor FMT in treating TKI-induced diarrhoea
- To investigate changes in gut microbiome after treatment in patients treated with donor FMT

3. METHODS

3.1 Study design

Single-centre placebo-controlled, double blind randomised clinical trial of donor FMT vs placebo FMT in patients with TKI-induced diarrhoea

3.2 Study population

Patients will be recruited among those referred to the oncology outpatient clinic of the Fondazione Policlinico Universitario "A. Gemelli". Patients with all inclusion criteria and none of the exclusion criteria will be considered for this study.

3.2.1 Inclusion criteria

- 18 years old or older
- Treatment with pazopanib or sunitinib for metastatic RCC diagnosed at histology and measurable according to RECIST criteria version 1.1 (23)
- Development of diarrhoea of 2-3 grade according to Common Terminology Criteria (CTC) for Adverse Events (AE) version 4.0 (24) induced by these drugs.
- Execution of a CT scan no earlier than 4 weeks before enrollment
- Good or intermediate prognostic assessment (according to criteria of the prognostic system of the International Metastatic RCC Database Consortium[25])
- Performance status equal or lower than 2 (26)
- Blood count, hepatic and kidney testing within normal limit
- Ability to give their consent to be included in the study.

3.2.2 Exclusion criteria

- Another known cause of diarrhoea (e.g. infectious gastroenteritis. *C. difficile* infection, celiac disease, inflammatory bowel disease, irritable bowel syndrome, chronic pancreatitis, biliary salt diarrhoea)
- Previous colorectal surgery or cutaneous stoma
- Food allergies
- Recent (<6 weeks) therapy with drugs that could possibly alter gut microbiota (e.g. antibiotics, probiotics, proton pump inhibitors, immunosuppressants, metformin)
- Another cancer (except for surgically treated basocellular carcinoma)
- Brain metastases
- Decompensated heart failure or heart disease with ejection fraction lower than 30%

- Severe respiratory insufficiency
- Psychiatric disorders
- Pregnancy
- Unable to give informed consent

Potentially eligible patients, based on these criteria, will undergo the following exams to exclude other causes of diarrhoea:

- Faecal exams, including: *C. difficile* (culture and toxin); bacterial culture for enteric pathogens, including *Salmonella*, *Shigella*, *Campylobacter*, *Escherichia coli* O157 H7, *Yersinia*, VRE (vancomycin-resistant Enterococci), MRSA (methicillin-resistant *Staphylococcus aureus*), Gram-negative MDR (multi-drug-resistant) bacteria, *Vibrio cholerae*, *Listeria monocytogenes*; Norovirus; Protozoa and helminths/Ova and parasites; faecal pancreatic elastase;
- Blood exams, including: C reactive protein, erythrocyte sedimentation rate, transglutaminase antibodies, total IgA and IgE
- Ileocolonoscopy

All subjects who will meet eligibility criteria and will test negative for these exams will be finally enrolled in the study.

3.3 Baseline assessments

Before randomisation, demographic data will be collected by the oncology staff, and patients will be evaluated for the severity of diarrhoea according to the National Cancer Institute Common Toxicity Criteria; NCI CTC version 4.0 (grade 0 = none; grade 1 = increase of < 4 stools/day over pre-treatment; grade 2 = increase of 4-6 stools/day, or nocturnal stools; grade 3 = increase of ≥ 7 stools/day or incontinence or need for parenteral support for dehydration; grade 4 = physiologic consequences requiring intensive care, or hemodynamic collapse) (24).
Additionally, patients will be requested to give stool samples to be collected in a sterile, sealed container and stored at -80°C for metagenomic assessment of gut microbiome by the microbiology staff.

3.4 Treatments

After baseline assessments, patients will be randomly assigned to one of the following treatment arms:

- Donor FMT (D-FMT)
- Placebo FMT (P-FMT)

Patients in both groups will undergo a single FMT procedure by colonoscopy.
Each patient in the donor FMT group will receive faeces from one single donor.
Placebo FMT will be made of 250 mL water.
Loperamide will be allowed as anti-diarrhoeal medication if diarrhoea will not respond to experimental treatments.

3.4.1 Selection of stool donors

The selection of stool donors will be performed by the gastroenterology staff following protocols previously recommended by international guidelines (20), including:

1) A questionnaire to address donor medical history, including:

Infectious diseases

- History of, or known exposure to, HIV, HBV or HCV, syphilis, human T-lymphotropic virus I and II, malaria, trypanosomiasis, tuberculosis
- Known systemic infection not controlled at the time of donation
- Use of illegal drugs
- Risky sexual behavior (anonymous sexual contacts; sexual contacts with prostitutes, drug addicts, individuals with HIV, viral hepatitis, syphilis; work as prostitute; history of sexually transmittable disease)
- Previous reception of tissue/organ transplant
- Previous (<12 months) reception of blood products
- Recent (<6 months) needle stick accident
- Recent (<6 months) body tattoo, piercing, earring, acupuncture
- Recent medical treatment in poorly hygienic conditions
- Risk of transmission of diseases caused by prions
- Recent parasitosis or infection from rotavirus, *Giardia lamblia* and other microbes with gastrointestinal involvement
- Recent (<6 months) travel in tropical countries, countries at high risk of communicable diseases or traveller's diarrhoea

- Recent (<6 months) history of vaccination with a live attenuated virus, if there is a possible risk of transmission
- Healthcare workers (to exclude the risk of transmission of multidrug-resistant organisms)
- Individual working with animals (to exclude the risk of transmission of zoonotic infections)

Gastrointestinal, metabolic and neurological disorders

- History of irritable bowel syndrome, inflammatory bowel disease, functional chronic constipation, coeliac disease, other chronic gastrointestinal disorders
- History of chronic, systemic autoimmune disorders with gastrointestinal involvement
- History of, or high risk for, gastrointestinal cancer or polyposis
- Recent appearance of diarrhoea, hematochezia
- History of neurological/neurodegenerative disorders
- History of psychiatric conditions
- Overweight and obesity (body mass index >25)

Drugs that can impair gut microbiota composition

- Recent (<3 months) exposure to antibiotics, immunosuppressants, chemotherapy
- Chronic therapy with proton pump inhibitors

2) Blood and stool exams to exclude potentially transmittable diseases, including:

Blood exams

- Cytomegalovirus
- Epstein-Barr virus
- Hepatitis A
- HBV
- HCV
- Hepatitis E virus
- Syphilis
- HIV-1 and HIV-2
- *Entamoeba histolytica*
- Complete blood cell count with differential
- C-reactive protein and erythrocyte sedimentation rate

- ▸ Albumin
- ▸ Creatinine and electrolytes
- ▸ Aminotransferases, bilirubin, gamma-glutamyltransferase, alkaline phosphatase

Stool exams

▸ Detection of *C. difficile*

▸ Detection of enteric pathogens, including *Salmonella*, *Shigella*, *Campylobacter*, *Escherichia coli O157 H7*, *Yersinia*, vancomycin-resistant Enterococci, methicillin-resistant *Staphylococcus aureus*, Gram-negative multidrug-resistant bacteria

▸ Norovirus

▸ Antigens for *Giardia lamblia* and *Criptosporidium parvum*

▸ Protozoa (including *Blastocystis hominis*) and helminths

▸ Faecal occult blood testing

▸ Calprotectin

▸ *Helicobacter pylori* faecal antigen

▸ Rotavirus

3) A further questionnaire administered to selected donors the day of the faeces collection to rule out any issue happened within the screening period, including:

▸ Newly appeared gastrointestinal signs and symptoms (i.e. diarrhoea, nausea, vomiting, abdominal pain, jaundice)

▸ Newly appeared illness or general signs as fever, throat pain, swollen lymph nodes

▸ Use of antibiotics or other drugs that may impair gut microbiota, new sexual partners or travels abroad since the last screening

▸ Recent ingestion of a substance that may result harmful for the recipients

▸ Travel in tropical areas—contact with human blood (sting, wound, showing, piercings, tattoos)—sexual high-risk behaviour

▸ Diarrhoea (more than three loose or liquid stools per day) among members of the entourage (including children) within 4 weeks of donation

The assignment of faecal infusates from healthy donors to patients will be done randomly, without any specific recipient-donor match, as this is not recommended by international guidelines (20).

Two healthy subjects will be selected, among donors from the donor bureau of the gastroenterology unit, as stool donors. At the time of their first donation, a sample of faeces will be collected and stored at -80°C for microbiome analysis.

3.4.2 Manufacturing of faecal infusates

All faecal infusates will be manufactured in the microbiology unit of our hospital. Only fresh faeces will be used. For each aliquot, 50 grams of faeces will be diluted in 250 mL of sterile saline. The deriving solution will be blended, and the supernatant strained and poured into a sterile container.

3.4.3 FMT procedures

All procedures will be performed by colonoscopy, under sedation. Patients in both groups wil undergo bowel cleansing with 4 litres of macrogol (SELG ESSE) the day before the procedure. All procedures will be performed by 2 expert endoscopists (G. C., L. R. L.), using pediatric colonoscopes and carbon dioxide insufflation. Both faecal infusates and placebo infusates will be delivered through the operative channel of the scope after reaching the more proximal point of the large bowel, using 50 mL syringes filled with the infusate during colonoscopy. The faecal infusate will be delivered within 6 hours after donor supply. After the procedures, patients will be monitored in the recovery room of the endoscopy centre for nearly 3 hours.

3.5 Follow-up

Follow-up visits will be performed by physicians from the oncology unit. All patients will be followed up for 2 months after the end of treatments. Follow-up visits will be scheduled at week 1, week 2, week 4, and week 8, after the end of treatments, respectively. At each visit the following assessments will be performed: 1) evaluation of the severity of diarrhoea, following the National Cancer Institute Common Toxicity Criteria (NCI CTC) version 4.0 (24); 2) record of concomitant medication, including loperamide; 3) collection of stool samples; 4) record of adverse events. Unscheduled follow-up visits will be offered if requested by the patients.

3.6 Study Outcomes

3.6.1 Primary outcome

- Resolution of diarrhoea 4 weeks after the end of treatments

3.6.2 Secondary outcomes

- Resolution of diarrhoea 1, 2, and 8 weeks after the end of treatments
- Decrease of diarrhoea until grade G1 or lower 1, 2, 4 and 8 weeks after the end of treatment
- Discontinuation or reduction of treatment with TKIs

All adverse event occurred during follow-up will be recorded

3.7 Randomization and treatment allocation

Blocked randomisation of subjects will be performed by an external individual not involved in the study. An online random number generator software (https://www.sealedenvelope.com/simple-randomise r/v1/lists) will be used. To mask treatments to recipients, both infusates bottles and syringes will be covered with dark colored paper before the infusion, and the patients will be unable to see the endoscopic display during the procedure, which will be done under sedation. Moreover, the physicians who will visit patients at follow-up will be not aware of the treatment being administered.

3.8 Gut microbiota analysis

Gut microbiota analysis will be performed via shotgun metagenomics. Whole DNA will be extracted with the DNA was extracted using the Danagene Microbiome Fecal DNA kit and sequenced on the Illumina NovaSeq platform at an average of at least 4.5Gb. Default quality control will be done following the recommended and validated bioBarkery workflow, and resulting samples analyzed with the latest releases of MetaPhlAn for taxonomic profiling and StrainPhlAn for strain-level profiling.

3.9 Sample size

To calculate sample size, we assume a 20% resolution rate of diarrhoea in the placebo arm (21) and a 80% resolution rate of diarrhoea in the FMT arm at 4 weeks of follow-up. Using a two-tailed a value of 0.05 and a power of 80% (b = 0.20), the enrolment of 10 patients per group is required.

3.10 Statistical analysis

The statistical analysis will be performed both on an intention-to-treat and per-protocol basis. Differences among groups will be assessed with a two-tailed Wilcoxon-rank sum test for

continuous data and with Fisher's exact probability test (using two-tailed P-values) for categorical data. Differences in cure percentages will be determined with Fisher's exact test (with two-tailed P values). For microbiome analysis, statistical differences between group means will be calculated using a two-tailed Wilcoxon-Rank Sum Test, through the R statistical software package (R Core Team, Vienna, Austria).

4. Safety Reporting

No specific serious adverse events are expected. Adverse events are defined as any undesirable experience occurring to a subject during the study, whether or not considered related to the surveillance protocol. All adverse events reported spontaneously by the subject or observed by the investigator or his staff will be recorded and reported to the coordinating investigator.

A serious adverse event is any untoward medical occurrence or effect that at any dose:

- results in death;
- is life threatening (at the time of the event);
- requires hospitalisation or prolongation of existing inpatients' hospitalisation;
- results in persistent or significant disability or incapacity;
- is a congenital anomaly or birth defect;
- is a new event of the trial likely to affect the safety of the subjects, such as an unexpected outcome of an adverse reaction, lack of efficacy of an IMP used for the treatment of a life threatening disease, major safety finding from a newly completed animal study, etc.

All SAEs will be reported by the coordinating investigator.
SAEs that result in death or are life threatening should be reported expedited. The expedited reporting will occur not later than 7 days after the responsible investigator has first knowledge of the adverse reaction. This is for a preliminary report with another 8 days for completion of the report.
All adverse events will be followed until they have abated, or until a stable situation has been reached. Depending on the event, follow up may require additional tests or medical procedures as indicated, and/or referral to the general physician or a medical specialist.

5. Ethics

The study protocol must be approved by the ethics committee of the Fondazione Policlinico Universitario "A. Gemelli" IRCCS, and will be registered at ClinicalTrials.gov. The study will be conducted in accordance with the Consolidated Standards of Reporting Trials (CONSORT) Statement

6. References

1. Siegel RL, Miller KD, Jemal A. Cancer statistics, 2018. CA Cancer J Clin 2018;68:7–30.
2. http://www.cancerresearchuk.org/health-professional/cancer-statistics/statistics-by-cancer-type/kidney-cancer/incidence-heading-Zero.
3. Capitanio U, Bensalah K, Bex A, Boorjian SA, Bray F, Coleman J, Gore JL, Sun M, Wood C, Russo P. Epidemiology of Renal Cell Carcinoma. Eur Urol. 2019;75:74-84.
4. Capitanio U, Montorsi F. Renal cancer. Lancet 2016;387:894–906.
5. Surveillance, Epidemiology, and End Results Program. SEER stat fact sheets: kidney and renal pelvis cancer. Bethesda, MD: National Cancer Institute (http://seer.cancer .gov/ statfacts/ html/ kidrp .html).
6. Ferlay J, Soerjomataram I, Ervik M, et al. GLOBOCAN 2012 v1.0, Cancer incidence and mortality worldwide: IARC CancerBase No.11. Lyon, France: International Agency for Research on Cancer; 2013. http://globocan.iarc.fr.
7. Rao A, Wiggins C, Lauer R. Survival outcomes for advanced kidney cancer patients in the era of targeted therapies. Ann Transl Med. 2018;6:165.
8. Choueiri TK, Motzer RJ. Systemic Therapy for Metastatic Renal-Cell Carcinoma. N Engl J Med. 2017;376:354-366.
9. Gore M, Szczylik C, Porta C, Bracarda S, Bjarnason G, Oudard S, et al. Final results from the large sunitinib global expanded-access trial in metastatic renal cell carcinoma. Br J Cancer 2015; 113:12–19.
10. Sternberg CN, Davis ID, Mardiak J, Szczylik C, Lee E, Wagstaff J, et al. Pazopanib in locally advanced or metastatic renal cell carcinoma: results of a randomized phase III trial. J Clin Oncol 2010;28:1061–8.
11. Motzer RJ, Hutson TE, Tomczak P, Michaelson MD, Bukowski RM, Rixe O, et al. Sunitinib versus interferon alfa in metastatic renal-cell carcinoma. N Engl J Med 2007;356:115–24.
12. Jonasch E, Slack RS, Geynisman DM, Hasanov E, Milowsky MI, Rathmell WK, Stovall S, Juarez D, Gilchrist TR, Pruitt L, Ornstein MC, Plimack ER, Tannir NM, Rini BI. Phase II Study of Two Weeks on, One Week off Sunitinib Scheduling in Patients With Metastatic Renal Cell Carcinoma. J Clin Oncol. 2018;36:1588-1593.
13. Appleby L, Morrissey S, Bellmunt J, Rosenberg J. Management of treatment-related toxicity with targeted therapies for renal cell carcinoma: evidence-based practice and best practices. Hematol Oncol Clin North Am 2011;25:893–915

14. Montassier E, Gastinne T, Vangay P, Al-Ghalith GA, Bruley des Varannes S, Massart S, Moreau P, Potel G, de La Cochetière MF, Batard E, Knights D. Chemotherapy-driven dysbiosis in the intestinal microbiome. Aliment Pharmacol Ther. 2015;42:515-28.
15. Boers-Sonderen MJ, Mulder SF, Nagtegaal ID, Derikx LA, Wanten GJ, Mulders PF, van der Graaf WT, Hoentjen F, van Herpen CM. Endoscopy in patients with diarrhoea during treatment with vascular endothelial growth factor receptor tyrosine kinase inhibitors: Is the cause in the mucosa? Acta Oncol. 2016;55(4):444-8.
16. Pal SK, Li SM, Wu X, Qin H, Kortylewski M, Hsu J, Carmichael C, Frankel P. Stool Bacteriomic Profiling in Patients with Metastatic Renal Cell Carcinoma Receiving Vascular Endothelial Growth Factor-Tyrosine Kinase Inhibitors. Clin Cancer Res. 2015;21:5286-93.
17. Chitapanarux I, Chitapanarux T, Traisathit P, Kudumpee S, Tharavichitkul E, Lorvidhaya V. Randomized controlled trial of live lactobacillus acidophilus plus bifidobacterium bifidum in prophylaxis of diarrhoea during radiotherapy in cervical cancer patients. Radiat Oncol. 2010;5:31.
18. Wei D, Heus P, van de Wetering FT, van Tienhoven G, Verleye L, Scholten RJ. Probiotics for the prevention or treatment of chemotherapy- or radiotherapy-related diarrhoea in people with cancer. Cochrane Database Syst Rev. 2018 Aug 31;8:CD008831.
19. Ianiro G, Maida M, Burisch J, Simonelli C, Hold G, Ventimiglia M, Gasbarrini A, Cammarota G. Efficacy of different faecal microbiota transplantation protocols for Clostridium difficile infection: A systematic review and meta-analysis. United European Gastroenterol J. 2018;6:1232-1244
20. Cammarota G, Ianiro G, Tilg H, Rajilić-Stojanović M, Kump P, Satokari R, Sokol H, Arkkila P, Pintus C, Hart A, Segal J, Aloi M, Masucci L, Molinaro A, Scaldaferri F, Gasbarrini G, Lopez-Sanroman A, Link A, de Groot P, de Vos WM, Högenauer C, Malfertheiner P, Mattila E, Milosavljević T, Nieuwdorp M, Sanguinetti M, Simren M, Gasbarrini A; European FMT Working Group. Gut. 2017;66:569-580.
21. Rossen NG, Fuentes S, van der Spek MJ, Tijssen JG, Hartman JH, Duflou A, Löwenberg M, van den Brink GR, Mathus-Vliegen EM, de Vos WM, Zoetendal EG, D'Haens GR, Ponsioen CY. Findings From a Randomized Controlled Trial of Fecal Transplantation for Patients With Ulcerative Colitis. Gastroenterology. 2015;149:110-118.e4.
22. Kootte RS, Levin E, Salojärvi J, Smits LP, Hartstra AV, Udayappan SD, Hermes G, Bouter KE, Koopen AM, Holst JJ, Knop FK, Blaak EE, Zhao J, Smidt H, Harms AC, Hankemeijer T, Bergman JJGHM, Romijn HA, Schaap FG, Olde Damink SWM, Ackermans MT, Dallinga-Thie GM, Zoetendal E, de Vos WM, Serlie MJ, Stroes ESG, Groen AK, Nieuwdorp M. Improvement

of Insulin Sensitivity after Lean Donor Feces in Metabolic Syndrome Is Driven by Baseline Intestinal Microbiota Composition. Cell Metab. 2017;26:611-619.e6.

23. Eisenhauer EA, Therasse P, Bogaerts J, Schwartz LH, Sargent D, Ford R, Dancey J, Arbuck S, Gwyther S, Mooney M, Rubinstein L, Shankar L, Dodd L, Kaplan R, Lacombe D, Verweij J. New response evaluation criteria in solid tumours: revised RECIST guideline (version 1.1) Eur J Cancer. 2009;45:228–47.
24. (https://ctep.cancer.gov/protocolDevelopment/electronic_applications/ctc.htm#ctc_40)
25. Heng DY, Xie W, Regan MM, Warren MA, Golshayan AR, Sahi C, Eigl BJ, Ruether JD, Cheng T, North S, Venner P, Knox JJ, Chi KN, Kollmannsberger C, McDermott DF, Oh WK, Atkins MB, Bukowski RM, Rini BI, Choueiri TK. Prognostic factors for overall survival in patients with metastatic renal cell carcinoma treated with vascular endothelial growth factor-targeted agents: results from a large, multicenter study. J Clin Oncol. 2009;27:5794-9.
26. Oken MM, Creech RH, Tormey DC, Horton J, Davis TE, McFadden ET, Carbone PP. Toxicity and response criteria of the Eastern Cooperative Oncology Group. Am J Clin Oncol. 1982;5:649-55.
