# Supplementary material for: Faecal microbiota transplantation for the treatment of diarrhoea induced by tyrosine-kinase inhibitors in patients with metastatic renal cell carcinoma
Source: Nat Commun. 2020 Aug 28;11:4333. doi: 10.1038/s41467-020-18127-y (PMC7455693; doi:10.1038/s41467-020-18127-y)
Supplement: Supplementary file 3 — Reporting Summary [file 41467_2020_18127_MOESM3_ESM.pdf]

nature research

Corresponding author(s): Giovanni Cammarota
Last updated by author(s): 05 August 2020

Reporting Summary

Nature Research wishes to improve the reproducibility of the work that we publish. This form provides structure for consistency and transparency in reporting. For further information on Nature Research policies, seeAuthors & Referees and theEditorial Policy Checklist .

Statistics

For all statistical analyses, confirm that the following items are present in the figure legend, table legend, main text, or Methods section.

n/a	Confirmed	
[ ]	[x]	The exact sample size (*n*) for each experimental group/condition, given as a discrete number and unit of measurement
[ ]	[x]	A statement on whether measurements were taken from distinct samples or whether the same sample was measured repeatedly
[ ]	[x]	The statistical test(s) used AND whether they are one- or two-sided<br>*Only common tests should be described solely by name; describe more complex techniques in the Methods section.*
[x]	[ ]	A description of all covariates tested
[ ]	[x]	A description of any assumptions or corrections, such as tests of normality and adjustment for multiple comparisons
[ ]	[x]	A full description of the statistical parameters including central tendency (e.g. means) or other basic estimates (e.g. regression coefficient) AND variation (e.g. standard deviation) or associated estimates of uncertainty (e.g. confidence intervals)
[ ]	[x]	For null hypothesis testing, the test statistic (e.g. *F*, *t*, *r*) with confidence intervals, effect sizes, degrees of freedom and *P* value noted<br>*Give P values as exact values whenever suitable.*
[x]	[ ]	For Bayesian analysis, information on the choice of priors and Markov chain Monte Carlo settings
[x]	[ ]	For hierarchical and complex designs, identification of the appropriate level for tests and full reporting of outcomes
[x]	[ ]	Estimates of effect sizes (e.g. Cohen's *d*, Pearson's *r*), indicating how they were calculated

Our web collection on statistics for biologists contains articles on many of the points above.

Software and code

Policy information about availability of computer code

Data collection	Microsoft Excel for Mac (Microsoft Excel. Redmond, Washington: Microsoft, 2011).<br>"MetaPhlAN 3.0 is available in GitHub at https://github.com/biobakery/MetaPhlAn/tree/3.0<br>StrainPhlAN 3.0 is available in GitHub at https://github.com/biobakery/MetaPhlAn/wiki/StrainPhlAn-3.0<br>The metagenome pre-processing script is available in GitHub at https://github.com/SegataLab/preprocessing<br>bowtie2 (version 2.3.4.3)<br>trim_galore (version 0.5.0)"
Data analysis	Statistical analyses of clinical data were performed through an online calculator (http://www.graphpad.com/quickcalcs/);

For manuscripts utilizing custom algorithms or software that are central to the research but not yet described in published literature, software must be made available to editors/reviewers. We strongly encourage code deposition in a community repository (e.g. GitHub). See the Nature Research guidelines for submitting code & software for further information.

Data

Policy information about availability of data

All manuscripts must include a data availability statement. This statement should provide the following information, where applicable:

- Accession codes, unique identifiers, or web links for publicly available datasets
- A list of figures that have associated raw data
- A description of any restrictions on data availability

The software code for the study of the metagenomic data is open source and available inside the MetaPhlAn repository (https://github.com/biobakery/MetaPhlAn) under tag 3.0. Nucleotide sequences are available in the Sequence Read Archive under the bioproject accession PRJNA643802.
Clinical data of individual patients will be shared, after proper de-identification, upon reasonable request to the corresponding author from colleagues who want to analyse in deep our findings, from now to the next 3 years.

Field-specific reporting

Please select the one below that is the best fit for your research. If you are not sure, read the appropriate sections before making your selection.

[x] Life sciences [ ] Behavioural & social sciences [ ] Ecological, evolutionary & environmental sciences

For a reference copy of the document with all sections, see nature.com/documents/nr-reporting-summary-flat.pdf

Life sciences study design

All studies must disclose on these points even when the disclosure is negative.

Sample size	To calculate sample size, we assumed:<br>- a 20% resolution rate of diarrhoea in the placebo arm, as observed in the placebo-controlled randomised trial of FMT in ulcerative colitis published by Rossen et al, Gastroenterology 2015). Although other randomised trials already published a the time of the beginning of our study (e.g. Moayyedi et al, Gastroenterology 2015 or Paramsothy et al, Lancet 2017) observed a lower rate of success in the placebo arm, we chose this one to be more conservative<br>- a 80% resolution rate of diarrhoea in the FMT arm. As there were no efficacy data of FMT in chemotherapeutics-dependent diarrhoea at the time of the beginning of our study, we considered 80% to be a reasonable resolution rate, given the success of FMT in C. difficile infection-associated diarrhoea (nearly 90%)<br>We assumed these rates to be achieved both after 4 weeks of follow-up.<br>Using a two-tailed a value of 0.05 and a power of 80% (b = 0.20), the enrolment of 10 patients per group was required.<br>Sample size was calculated with an online software (https://clincalc.com/).
Data exclusions	No data were excluded from the analysis
Replication	""Technical replication on metagenomic sequencing is not necessary and not performed in the metagenomic field. Biological replication would involve including new patients which is not appropriate given the sample size power calculation discussed above"
Randomization	Blocked randomisation of subjects was performed by an external individual not involved in the study. An online random number generator software (https://www.sealedenvelope.com/simple-randomise r/v1/lists) was used to provide random permuted blocks with a block size of four and an equal allocation ratio; the sequence was hidden until the interventions were assigned.
Blinding	To mask treatments to recipients, both bottles of faecal infusates and syringes were covered with dark colored paper before the infusion, and the patients were unable to see the endoscopic display during the procedure. Moreover, the physicians who evaluated patients at follow-up were not aware of the treatment being administered. So, only the microbiologists who prepared the infusates and the physicians who infused the material by colonoscopy were unblinded. We did not consider this unblinding relevant, as both patients and physicians who followed up patients after treatments were blinded, and the physicians who performed the procedure did not assess outcomes and did not perform analysis.

Reporting for specific materials, systems and methods

We require information from authors about some types of materials, experimental systems and methods used in many studies. Here, indicate whether each material, system or method listed is relevant to your study. If you are not sure if a list item applies to your research, read the appropriate section before selecting a response.

Materials & experimental systems

n/a	Involved in the study
x	Antibodies
x	Eukaryotic cell lines
x	Palaeontology
x	Animals and other organisms
	x Human research participants
	x Clinical data

Methods

n/a	Involved in the study
x	ChIP-seq
x	Flow cytometry
x	MRI-based neuroimaging

Human research participants

Policy information about studies involving human research participants

Population characteristics	Overall, we enrolled 20 subjects (F = 5, M = 15, mean age 65 years) who met inclusion and exclusion criteria, and have accepted to participate the study. Four subjects have been being treated with sunitinib and 16 subjects were receiving pazopanib during the study period. Nineteen patients presented with a G2 diarrhea, and one (in the D-FMT group) with a G3 diarrhea, according to the NCI CTC. Overall, there were no significant differences in demographic and clinical characteristics between the two groups at baseline
Recruitment	"All patients were recruited in the outpatient Comprehensive Cancer Centre of the Fondazione Policlinico Universitario "A. Gemelli" IRCCS. Patients. No potential recruitment biases were detected
Ethics oversight	"The study was approved by the Ethics Committee of the Fondazione Policlinico Universitario "A. Gemelli" IRCCS. The approval protocol number was 008098/19. All enrolled subjects gave their written informed consent to participate the study".

Note that full information on the approval of the study protocol must also be provided in the manuscript.

Clinical data

Policy information about clinical studies

All manuscripts should comply with the ICMJEguidelines for publication of clinical research and a completedCONSORT checklist must be included with all submissions.

Clinical trial registration	ClinicalTrials.gov Identifier: NCT04040712
Study protocol	The full study protocol is available upon request to the corresponding author
Data collection	Data were collected at the Policlinico Universitario "A. Gemelli" IRCCS, an academic tertiary care centre based in Rome, Italy, between August 2019 and December 2019
Outcomes	The primary outcome was resolution of diarrhoea 4 weeks after the end of treatments.<br>Secondary outcomes included: discontinuation or reduction of treatment with TKIs; resolution of diarrhoea 1, 2, and 8 weeks after the end of treatments, and decrease of diarrhoea until grade G1 or lower 1, 2, 4 and 8 weeks after the end of treatment, respectively. We recorded any adverse event occurred during follow-up. Outcomes were assessed at each follow-up visit
